# Sheep blastocyst-like structure models derived from stem cells

**DOI:** 10.1038/s41421-026-00872-x

**Published:** 2026-03-24

**Authors:** Jing Cao, Jie Li, Miaohan Jin, Wenwen Shen, Jie Li, Saizheng Han, Jiqiang Fu, Shengjiao Song, Shenshen Shang, Fei Gao, Bo Liu, Xi Cao, Xiaoyu Niu, Zhen Liu, Xiaolong Wang

**Affiliations:** 1 Hainan Institute of Northwest A&F University, Sanya, Hainan China; 2 College of Animal Science and Technology, Northwest A&F University, Yangling, Shaanxi China; 3 Institute of Neuroscience, CAS Center for Excellence in Brain Science and Intelligence Technology, CAS Key Laboratory of Primate Neurobiology, State Key Laboratory of Neuroscience, Chinese Academy of Sciences, Shanghai, China; 4 Ningxia Engineering Research Center for Tan Sheep Bio-Breeding, Wuzhong, Ningxia China; 5 Shanghai Center for Brain Science and Brain-Inspired Intelligence Technology, Shanghai, China

**Keywords:** Stem cells, Embryonic stem cells

Dear Editor,

In mice, it has been reported that co-culturing naïve embryonic stem cells (ESCs) with trophoblast cells can give rise to embryonic-like structures in vitro that closely resemble E3.5 blastocysts at both the morphological and transcriptional levels^1,2^. More recent research has focused on generating embryo models by combining two or three different cell types through the overexpression of specific transcription factors. For example, a mouse embryo model generated by assembling naïve ESCs, induced trophoblast stem cells (TSCs, iCDX2), and induced extraembryonic endoderm cells (XENs, iGATA6) is capable of recapitulating developmental stages up to approximately E8.5^3,4^, capturing key processes of post-gastrulation and early organogenesis.

In primates, ESCs differ from rodent ESCs in their differentiation potential. Human naïve ESCs can be induced to form blastoids as their ability to form the trophoblast lineage. Moreover, integrated embryo models have been established by combining ESCs with extraembryonic cells, successfully recapitulating key features of post-implantation human embryo^5,6^. These models provide an important platform for studying critical stages of human embryogenesis.

Stem cell-derived embryo models also hold great promise for improving breeding strategies in large animals. Bovine and porcine blastoids have been generated from ESCs using a three-dimensional (3D) differentiation protocol^7,8^. Despite sheep’s importance in wool and meat production, a pre-implantation blastocyst model has not yet been reported. Given that our sheep ESCs (sESCs) display characteristics closely resembling the primed state of primate ESCs^9^, we sought to establish a sheep blastoid model by applying a blastoid induction protocol with sESCs and sTSCs.

Prior to generating sheep extraembryonic cell lines, we first established sESC lines from blastocysts. The sESCs were successfully isolated and maintained long-term, exhibiting dome-shaped colonies with well-defined boundaries (Supplementary Figs. S1a, S2a). Even after more than 50 passages, the cells retained stable colony morphology without notable changes (Supplementary Fig. S1a) and exhibited high proliferative capacity (Supplementary Fig. S1b). Alkaline phosphatase (AP) staining confirmed robust AP activity in the established sESCs (Supplementary Fig. S1c).

Quantitative RT-PCR (RT-qPCR) and immunofluorescent staining confirmed the expression of key pluripotency marker, including SOX2, NANOG, OCT4 and SALL4 (Supplementary Fig. S1d, e). The differentiation potential of sESCs was assessed through embryoid body (EB) formation and teratoma assays, with H&E staining showing that sESCs could differentiate into all three germ layers (Supplementary Fig. S1f, g). Karyotype analysis of sESCs at passages 15 and 30 revealed normal chromosomal composition (Supplementary Fig. S1h). These findings collectively suggest that sESCs were well-maintained for a long term without differentiation under our culture conditions.

To identify factors that upregulate trophoblast gene expression, we first analyzed publicly available single-cell RNA sequencing (scRNA-seq) data from natural sheep embryos^10^. We observed high expression of *CDX2*, *GATA3*, and *WNT6* in the trophoblast cells (Supplementary Fig. S2b, c). Given that *CDX2* drives trophoblast lineage gene expression in both mouse and human ESCs^3,6^, we selected it as a candidate to promote the differentiation of sESCs into trophoblast-like cells.

To generate a transgenic sESC line, we introduced a doxycycline-inducible *CDX2* overexpression strategy and obtained the transgenic sESCs (Fig. 1a; Supplementary Fig. S2d). To optimize differentiation, we tested three doxycycline concentrations. After four days of induction, cells proliferated normally under all conditions (Supplementary Fig. S2e). RT-qPCR results showed that treatment with 1 μg/mL doxycycline had the highest efficiency in upregulating the expression of classical endogenous trophoblast genes (*CDX2*, *GATA3*, *KRT8* and *ASCL2*) compared with other groups (Fig. 1b). Therefore, we selected 1 μg/mL doxycycline for subsequent experiments. Immunofluorescence confirmed sustained trophoblast marker expression in long-term induced cells (Supplementary Fig. S2f). We further performed in vitro differentiation assays and found that the iCDX2-ESCs could differentiate into extravillous cytotrophoblasts (EVTs) and syncytiotrophoblasts (STs) following the established human protocol^11^ (Supplementary Fig. S3a), Immunofluorescence revealed that the EVT and ST lineages specifically expressed the EVT marker HLA-G and the ST marker SDC1, respectively **(**Supplementary Fig. S3b, d**)**. qPCR analysis further showed that several known markers of ST *(CGA*, *GCM1*, *CYP19A1*) and EVT (*CDH5*, *ITGA5*, *MMP2*) were significantly upregulated compared with iCDX2-ESCs cultured without doxycycline (Supplementary Fig. S3c, e). Collectively, these results indicate that iCDX2-sESCs closely resemble natural sTSCs.

**Fig. 1:**
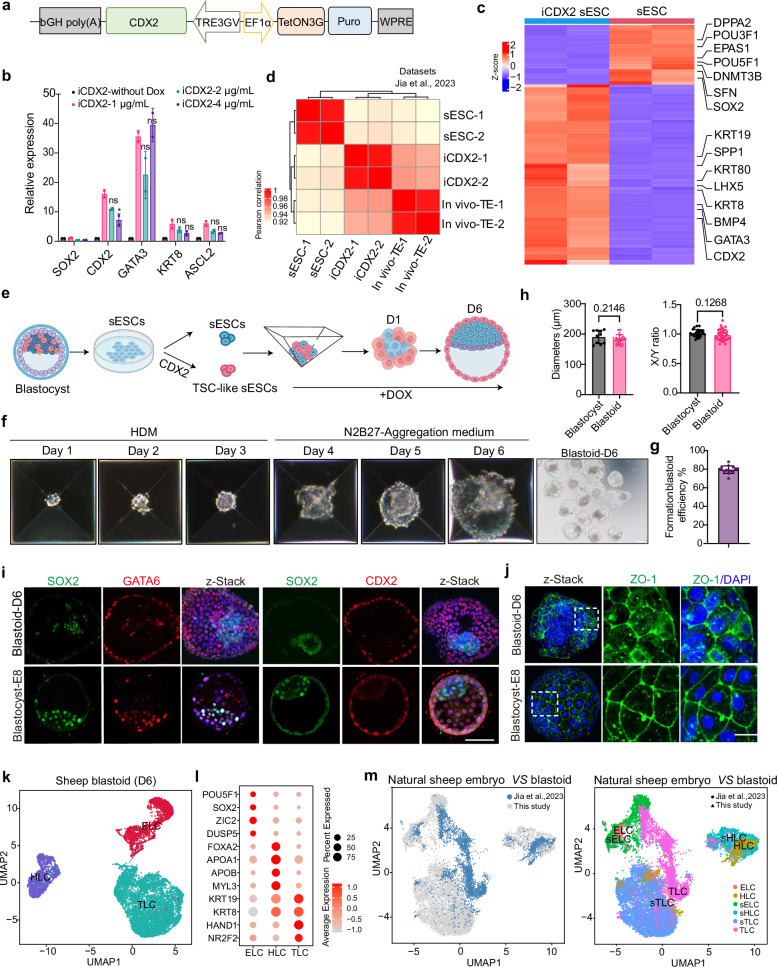
Generation of sheep blastoids from ESC and TSC cultures. **a** Generation of sESC with inducible overexpression of *CDX2*. **b** RT-qPCR for the indicated trophectoderm genes in inducible overexpression of *CDX2*. Doxycycline was added at concentrations of 1 µg/mL, 2 µg/mL, 4 µg/mL for 4 days. Data are presented as mean ± SEM of fold-change compared to primed ESCs. *P < 0.05. *n* = 3 biological replicates. **c** Heatmap showing the expression of pluripotency genes and trophectoderm genes in sESC and iCDX2-sESC (4 days of doxycycline addition in E8 + KSR medium). **d** Heatmap showing the similarity among sESC, iCDX2-sESC and in vivo trophectoderm (pseudobulk) from sheep blastocyst. TE trophectoderm. **e** Overview of the protocol for generating sheep blastoids by combining wild-type sESCs with iCDX2-sESCs, with 1 µg/mL doxycycline added throughout the entire culture period. **f** Representative images of cell aggregates at indicated time points during sheep blastoid formation. Scale bar, 50 μm. **g** Quantification of blastoid formation efficiency (*n* = 15). **h** Mean diameter (blastoids *n* = 30; blastocyst *n* = 10) and X/Y ratio (blastoids *n* = 35; blastocyst *n* = 27) of sheep blastoids and natural sheep E7–E8 blastocyst. Data are presented as mean ± SEM of fold-change. ns, *P* > 0.05. *n* = 3 biological replicates. **i** Representative immunofluorescent staining of markers of ELC (SOX2), TLC (CDX2) and HLC (GATA6) in sheep blastoids on D6. Scale bar, 50 μm. **j** Representative immunofluorescent staining of tight junction marker ZO-1 in sheep blastoid and natural blastocyst. Scale bar, 50 μm. **k** UMAP plot showing the major cluster classification of sheep blastoid, colored according to major cell types. **l** Dot plot indicating the expression of markers of ELC, HLC, and TLC. **m** UMAP plots depict the clustering of cells from sheep blastoids on D6 and spherical blastocytes on 9 days post-fertilization (dpf). ELC, HLC and TLC derived from spherical blastocytes; sELC, sHLC and sTLC derived from sheep blastoid.

To further investigate trophoblast gene expression, we performed bulk RNA-seq on iCDX2-sESC line (two biological replicates, Supplementary Fig. S4a, b). Analysis revealed progressive upregulation of trophoblast-enriched genes, including *GATA3, KRT8*, and *CDX2*, while pluripotency genes like *OCT4, SOX2*, and *DNMT3B* were downregulated compared to control sESCs (Fig. 1c; Supplementary Fig. S4c). To evaluate the transcriptional similarity between iCDX2-sESCs and natural sheep trophoblast cells, we integrated these data with previously published RNA-seq datasets from sheep blastocysts^10^. Correlation analysis showed that iCDX2-sESCs clustered closely with sheep trophoblast cells, indicating high gene expression similarity (Fig. 1d). Furthermore, differentially expressed gene (DEG) analysis identified several upregulated genes, including *ANXA8* and *KRT80* (Supplementary Fig. S4d). Gene Ontology (GO) analysis of the upregulated genes revealed enrichment in terms related to biological regulation and developmental processes (Supplementary Fig. S4e). Additionally, upregulated genes also were enriched in signaling pathways such as the PI3K-Akt signaling pathway and pathways regulating pluripotency (Supplementary Fig. S4f). These findings suggest that iCDX2-sESCs closely resemble trophoblast cells of sheep pre-implantation embryo.

To investigate whether the combination of iCDX2-sESCs and sESCs could generate blastoids in vitro, we co-cultured these two types of cells in a 3D system using sequential media changes^1^. Based on previous studies in mice and human, we tested various aggregation culture medium to identify optimal conditions for blastoid formation. Briefly, sESCs and iCDX2-sESCs were dissociated into single cells and mixed at a 1:3 ratio before being aggregated in an Aggrewell plate (Fig. 1e). After 24 h of aggregation, the aggregated cells were switched to N2B27 medium supplemented with doxycycline (referred to as AC-#1). However, by 5–6 days post-aggregation, most cells either died or formed spheroid structures in the 3D culture (Supplementary Fig. S5a, b). Given that the differentiation of hypoblast-like cells (HLCs) plays a key role in supporting blastoid formation, we hypothesized that the medium was insufficient to support HLC differentiation and the proliferation of aggregated cells. To address this, we applied a two-step induction protocol^12^. Initially, the cell mixture was cultured in hypoblast differentiation medium (HDM) containing FGF2, activin A, and CHIR99021 to promote HLC differentiation. After two days, compact cell aggregates formed. The medium was then switched to N2B27 for an additional 3–4 days, with doxycycline maintained throughout the entire culture period. We observed cavity-containing structures under the conditions (HDM-AC-#1); however, the frequency was low, and most of the blastoids were small (Supplementary Fig. S5a, b).

We then continued to improve the protocol to enhance the efficiency of sheep blastoid generation by adding a low dose of the chemical inhibitors to the N2B27 medium (referred to as AC-#2) during the second step of culture^12,13^. Under these optimized culture conditions, we observed a significant increase in the blastoid formation efficiency, with the presence of an inner cell mass (ICM)-like compartment and an outer trophectoderm-like layer (Supplementary Fig. S5b). We also tested the addition of the same inhibitors to the sESC culture medium (HDM-AC-#3) for an additional 3–4 days and a one-step induction method using modified N2B27 aggregate culture (AC-#2) for 5–7 days, but neither approach successfully generated cavity-containing blastoid structures (Supplementary Fig. S5a, b). We further optimized cell density and found that starting with ~25 cells yielded the highest blastoid formation efficiency (Supplementary Fig. S5c, d). This optimized condition, named N2B27-aggregate medium (HDM-AM-#2), successfully supported the formation of sheep blastoids with high efficiency (~80%) and with a structure resembling the blastocyst by day 6 (Fig. 1f, g). Morphologically, these blastoids resembled sheep blastocysts at E7–E8 (Supplementary Fig. S5e), and the diameter and X/Y ratio of blastoids were similar to those of sheep blastocysts (Fig. 1h). In summary, we developed an induction strategy that promotes the self-organization of sESCs and iCDX2-sESCs into blastoids, which exhibit characteristics closely resembling those of natural sheep blastocysts.

To investigate the characteristics of sheep blastoids, we conducted immunofluorescence analysis to detect markers expression of three cell lineages. Sheep blastoids exhibited lineage-specific marker expression, including *SOX2* for epiblast-like cells (ELCs), *GATA6* for HLCs, and *GATA3*, *KRT7*, and *CDX2* for trophectoderm-like cells (TLC) (Fig. 1i; Supplementary Fig. S6a). We quantified the numbers of SOX2^+^, GATA6^+^, and CDX2^+^ cells in both sheep blastocysts and blastoids. Although sheep blastoids contained more cells than E7 blastocysts, the proportions of these marker-positive cells were similar to those in the blastocysts (Supplementary Fig. S6b, c). Additionally, immunofluorescence staining for the tight junction marker ZO1 exhibited positive staining in the outer cells, consistent with the expression pattern in sheep blastocysts (Fig. 1j).

To further investigate the transcriptional characteristics of different cell lineages in the sheep blastoids, we conducted scRNA-seq analysis using day 6 blastoids (*n* = 9731 cells). We annotated 17 blastoid cell clusters into three major lineages based on known marker genes (Fig. 1k, l; Supplementary Fig. S7a–f)^10,14^. Further DEG and functional enrichment analysis revealed that ELC-specific genes were associated with regulation of transport and regulation of cell communication. HLC-specific genes were enriched in cell differentiation and lipid homeostasis; while TLC-specific genes were associated with embryonic placenta development and regulation of immune system process, suggesting an extra-embryonic fate (Supplementary Fig. S7d, e). Next, we integrated our blastoid data with published scRNA-seq data from natural sheep embryos^10,15^. UMAP visualization showed that ELC, TLC, and HLC in sheep blastoids closely resemble their counterparts in natural blastocysts, exhibiting transcriptomic characteristics highly similar to in vivo embryos (Fig. 1m; Supplementary Fig. S8a). Furthermore, correlation analysis revealed a strong similarity between the gene expression profiles of the sheep blastoids and pre-implantation embryos (Supplementary Figs. S7h and S8b, c). Collectively, this evidence supports that our in vitro-generated sheep blastoids recapitulate the key transcriptional features of their in vivo counterparts.

To verify the presence of functional ICM-like, hypoblast-like, and trophectoderm-like cells within sheep blastoids, we performed in vitro differentiation assays using individual day 6 (D6) blastoids to derive sESCs, sXENs, and sTSCs (Supplementary Fig. S9a). We successfully obtained and stably passaged sESCs and sXENs on feeder-based culture systems (Supplementary Fig. S9b). The blastoid-derived sESCs displayed a typical dome-shaped morphology and were positive for AP staining (Supplementary Fig. S9c). To further characterize these cells, we conducted qPCR and immunofluorescence analyses. qPCR and immunofluorescence staining results confirmed the lineage-specific expression patterns of sESC and sXEN markers (Supplementary Fig. S9d–f). However, due to the instability of the culture system, most blastoid-derived sTSCs did not survive beyond the third passage (data not shown).

To explore the potential of blastoids to grow and develop in 3D suspension culture, we selected D6 sheep blastoids with an ICM-like structure and a visible cavity for further prolonged in vitro culture (Supplementary Fig. S10a). These expanded blastoids continued to proliferate and grow up to day 18 in the shaker system (Supplementary Fig. S10b), with diameters ranging from 187 µm to 1809 µm in in vitro culture medium (Supplementary Fig. S10d). However, we observed a gradual decline in the survival rate of the blastoids over time, with 52% of the blastoids surviving on day 8, decreasing to 40% by day 10 and 10% by day 18 (Supplementary Fig. S10e). In contrast to blastoids, natural sheep blastocysts cultured under identical conditions exhibit higher survival rates and developmental capacity (Supplementary Fig. S10c, f). Notably, their survival rate remains ~63% by day 20 (Supplementary Fig. S10g). We subsequently performed immunofluorescence staining and observed the distinct expression of lineage markers, including SOX2, CDX2, and GATA6, localized to their expected positions (Supplementary Fig. S6h, i). These results further support the similarity between cultured blastoids and natural blastocysts, indicating that both can survive and expand during prolonged in vitro culture. Seasonal reproduction in sheep and technical constraints make in vivo transplantation challenging, necessitating future optimization of both in vitro culture and in vivo methodologies.

In summary, we first generated sTLC by doxycycline-induced *CDX2* expression, and this approach closely mimics trophoblast fate specification in sheep embryos. We further developed a method for generating sheep blastoid structures with a high efficiency (~80%) by combining wild-type sESCs with iCDX2-sESCs in a 3D induction system. The resulting blastoids exhibited morphology, molecular, and cell lineage features that closely resemble those of natural sheep blastocysts. Moreover, integrated analysis with published single-cell transcriptomes of natural sheep embryos confirmed that the sheep blastoids are transcriptionally similar. Additionally, both blastoids and natural blastocysts can survive and expand in vitro for over two weeks (Supplementary Fig. S11). This work represents a significant step forward in the development of livestock embryo models, providing a useful system for studying sheep embryogenesis.

## Supplementary information

Supplementary information

Supplementary Table S5

Supplementary Table S6

## Data Availability

The bulk RNA-seq dataset of sESCs and iCDX2-sESC and the raw scRNA-seq dataset of sheep blastoid generated in this study can be viewed in NODE (https://www.biosino.org/node) by pasting the accession number (OEP00006112) into the text search box or through the URL: https://www.biosino.org/node/project/detail/OEP00006112. The raw data of sheep spherical blastocyst (9 dpf) was downloaded from BioProject: PRJNA987334. The raw scRNA-seq data of natural sheep blastocytes (E7–E8) are downloaded from the Genome Sequence Archive (accession number: CRA018492).
