# Supplementary material for: Sheep blastocyst-like structure models derived from stem cells
Source: Cell Discov. 2026 Mar 24;12:20. doi: 10.1038/s41421-026-00872-x (PMC13013973; doi:10.1038/s41421-026-00872-x)
Supplement: Supplementary file 1 — Supplementary information [file 41421_2026_872_MOESM1_ESM.pdf]

Supporting Information for

Sheep blastocyst-like structure models derived from stem cells

Jing Cao[1,2,3,6], Jie Li[3,6], Miaohan Jin[2], Wenwen Shen[2], Jie Li[3], Saizheng Han[2], Jiqiang Fu[3], Shengjiao Song[2], Shenshen Shang[3], Fei Gao[3], Yanhua Guo[2], Bo Liu[2], Xi Cao[2], Xiaoyu Niu[5], Zhen Liu[3,4*], Xiaolong Wang[1,2*]

[1]Hainan Institute of Northwest A&F University, Sanya 572025, China

[2]College of Animal Science and Technology, Northwest A&F University, Yangling 712100, China

[3]Institute of Neuroscience, CAS Center for Excellence in Brain Science and Intelligence Technology, CAS Key Laboratory of Primate Neurobiology, State Key Laboratory of Neuroscience, Chinese Academy of Sciences, Shanghai 200031, China

[4]Shanghai Center for Brain Science and Brain-Inspired Intelligence Technology, Shanghai, China

[5]Ningxia Engineering Research Center for Tan Sheep Bio-Breeding, Wuzhong, 751999, China

[6]These authors contributed equally

*Correspondence: zliu2010@ion.ac.cn (Z.L.), xiaolongwang@nwafu.edu.cn (X.W)

This PDF file includes:

Materials and Methods
Supplementary References
Supplementary Figure
Supplementary Figure Legend
Supplementary Table S1-S6

Materials and Methods

Materials availability

This study did not generate any unique reagents.

Data availability

The bulk RNA-sea dataset of sheep ESCs and iCDX2-ESC and the raw scRNA-seq dataset of sheep blastoid generated in this study can be viewed in NODE (https://www.biosino.org/node) by pasting the accession (OEP00006112) into the text search box or through the URL: https://www.biosino.org/node/project/detail/ OEP00006112. The raw data of sheep spherical blastocyst (9 dpf) was downloaded from BioProject: PRJNA987334. The raw scRNA-seq data of sheep natural blastocytes (E7-E8) are downloaded from the Genome Sequence Archive (accession number: CRA018492).

Code availability

This paper does not report original code. Any additional information required to reanalyze the data reported in this paper is available from the lead contact upon request.

Feeders and fibroblast culture conditions

The MEF feeders and sheep fibroblast were cultured in DMEM (Gibco, 11995073) with 15% FBS (BI, 04-001-1ACS), 1 mM Glutamax (Gibco, 35050061), 1% penicillin-streptomycin (Gibco, 15140122), and 1% nonessential amino acids (Gibco, 11140050). All cell lines were tested and confirmed mycoplasma-free and were maintained in a 37°C incubator with 5% CO_2. Reagents used in this study in Supplementary Table S1.

Establishment of sESCs

In vivo-produced blastocysts were collected surgically on day 7. Briefly, the zona pellucida was removed using Tyrode's Acid solution (Sigma, T1788-100mL), followed by three washes using TePR [(mTeSRTM-Plus medium and 5 μM IWR (Sigma, I0161), also referred to as TePR]. The blastocysts were then plated onto 4-well plates containing Mitomycin C-treated mouse embryonic fibroblasts (MEF) in TePR culture conditions. Chang the fresh media every two days after the embryo attach the plate. The cells were cultured for one week until outgrowths became visible. At this point, the outgrowths were digested and passaged with TrypLE™ (Gibco, 2481905) onto fresh MEF feeders supplemented with 10 μM Y-27632 at a 1:1 ratio. Cell colonies were visible 1-2 days

after passaging. The media was replaced daily, and subsequent passaging occurred every 3-4 days at a 1:10 ratio. sESCs were passaged every 3-4 days, with the culture medium being refreshed daily. These cells were routinely cultured in E8+KOSR medium [(mTeSRTM-E8 medium; STEMCELL Technologies, 05990), 5% KOSR, and 2.5 μM IWR (Sigma, I0161), also referred to as E8+K]. All cell lines were confirmed to be mycoplasma-free and were maintained in an incubator at 37°C with 5% CO_2.

Transfection and generation of inducible *CDX2* sESCs lines

To generate Tet-on inducible lines, the lentiviral vector encoding the *CDX2* gene, pMD2.G and psPAX2 HEK293T cells were co-transfected into HEK293T cells using Lipofectamine 3000 (Invitrogen, L3000001) according to manufacturer's instructions. Briefly, HEK293T cells were cultured in basic medium (90% DMEM supplemented with 10% FBS), and the Lipofectamine 3000 reagent and plasmids were mixed in Opti-MEM medium (Gibco, 31985070). The transfection supernatant was collected twice, at 48 h and 72 h after transfection. The supernatant was filtered through a 0.45 μm filter to remove cell debris and clumps. Lentivirus particles were then concentrated by centrifuging the medium overnight at 12,000 rpm. To transduce sheep *CDX2* sESCs, the cells were dissociated into single cell using TrypLE and reseed in E8+K medium containing 10 μM ROCK inhibitor (Y27632). After 24 hours, the sESCs were transduced by incubating with lentiviral particles and polybrene for 24 hours in fresh E8+K medium. Two days after transfection, 2 μg/mL puromycin was added for 48-72 hours to select transduced *CDX2* sESCs clones, which were then reseeded onto fresh ICR feeder layers. Single cell sESC were passaged every 3-4 days. *CDX2* positive sESCs clones were selected, dissociated, and expanded in E8+K medium. Transgene activation was initiated by the addition of doxycycline hyclate. To induce transgene activation, doxycycline hyclate was added. To determine the optimal doxycycline concentration for subsequent experiments, we tested 1 μg/mL, 2 μg/mL, and 4 μg/mL doxycycline by adding it to the medium for 4 days, followed by quantitative PCR analysis of the collected cells.

Generation of sheep blastoids

For sheep blastoid formation, sESCs and iCDX2 sESCs were washed with PBS and dissociated into single cells by TrypLE Express at 37 °C for 5 min. Cells were collected and resuspended after centrifugation at 300 g for 5 min. The supernatant containing sESCs and *CDX2* sESCs were

collected and passed through a 40 μm cell strainer and counting the cells. Meanwhile, the AggreWell 400 (STEMCELL Technologies) was precoated with 1mL of Anti-Adherence Rinsing Solution (Stemcell technologies) and spun for 5 minutes at 2,000g, and incubated at room temperature for 10 min. After incubation, wells were washed with 1mL culture medium once. Current protocol is optimized for approximately 7500 sheep ESCs and 22500 *CDX2* positive ESCs cells in seeded into one well of a prepared AggreWell 400 24-well plate (around 25 cells per microwell,1:3 rates). And were resuspended in sheep ESCs medium supplemented with 5 μM Y-27632. The plate was centrifuged at 200g for 1 min and placed in a 37 °C with 5% CO2 and 5% Oxygen incubator. The medium was replaced with HDM 12 hrs later, designated as day 1. On day 3, carefully remove as much HDM as possible, and then add 1 mL of aggregation medium. Repeat this step one additional time to help completely remove the remaining HDM. Fresh aggregation medium is replaced every two days. After five or six days of culture in aggregation medium, sheep blastoids can be observed in some microwells. All formed blastoids were manually isolated using a mouth pipette under a stereomicroscope for downstream experiments.

The HDM was prepared using the following: 1:1 (v/v) mixture of DMEM/F12 and neurobasal medium, 1 x N2 supplement, 1 x B27 supplement, 1 x GlutaMAX, 1 x nonessential amino acids, 0.1 mM β-mercaptoethanol, 0.5% penicillin/streptomycin, 20 ng/mL bFGF (Peprotech), 20 ng/mL Activin A and 3 μM CHIR99021.

The different aggregation medium (AM) was prepared using the following:

N2B27 basic medium (AC-#1): 1:1 (v/v) mixture of DMEM/F12 and neurobasal medium, 0.5 x N2 supplement, 0.5 x B27 supplement, 1x GlutaMAX, 1x nonessential amino acids, 1% penicillin–streptomycin, 1 x Na-pyruvate, 0.1 mM β-mercaptoethanol, 1.6 mg/mL BSA and 1 μg/mL doxycycline.

N2B27 basic aggregate culture (AC-#2): 1:1 (v/v) mixture of DMEM/F12 and neurobasal medium, 0.5 x N2 supplement, 0.5 x B27 supplement, 0.5% ITS-X, 0.5 x GlutaMAX, 0.5 x nonessential amino acids, 0.5% penicillin–streptomycin, 0.5 x Na-pyruvate, 0.05 mM β-mercaptoethanol, 0.5% knockout serum replacement (KSR, Gibco), 0.1% FBS, 0.5 μM PD0325901, 0.5 μM A83-01, 1 μM CHIR99021, 0.5 μM SB431542, 25 ng/mL EGF, 0.75 μg/mL l-ascorbic acid and 0.4 mM VPA, 1.6 mg/mL BSA and 1 μg/mL doxycycline.

sESCs culture medium basic aggregate culture (AC-#3): 1:1 (v/v) mixture of DMEM/F12 and neurobasal medium, E8 medium, 0.5% ITS-X, 2.5% KOSR, and 1.7 μM IWR ,0.5% penicillin–streptomycin, 0.05 mM β-mercaptoethanol, 0.1% FBS, 0.5 μM PD0325901, 0.5 μM A83-01, 1 μM CHIR99021, 0.5 μM SB431542, 25 ng/mL EGF, 0.75 μg/mL L-ascorbic acid and 0.4 mM VPA, 1.6 mg/mL BSA and 1 μg/mL doxycycline.

Quantification of blastoid formation efficiency

Cell aggregates were collected from microwells by gently pipetting up and down 1–2 times with a 1mL pipette. To minimize the shearing force, around 1 mm from the end of the 1mL pipette tips were cut off before using. All the cell aggregates were transferred into a well of a six-well plate and counted under a stereomicroscope. The aggregates with the presence of an ICM-like compartment, a trophectoderm-like compartment and a visible cavity were counted as blastoids. The blastoid formation efficiency was calculated as the number of blastoids per number of total aggregates.

Derivation of stem cell lines from sheep blastoids

The previously described protocols to derive and culture sESCs cells and sXENs[1]. In brief, individual sheep blastoids were transferred onto mitomycin C-treated mouse embryonic fibroblast feeder in 3.5cm dish and cultured in E8+K medium (for blastoid-sESCs), NACL medium (for blastoid-sXENs). Outgrowths could be observed within 1 week. The blastoid will attach the dish around 24h, outgrowths could be observed within 1 week and dissociated with TrypLE Express and passaged onto fresh prepared feeder dish. Individual colonies were dissociated every 3-4 days. Cultures were maintained at 37 °C, 5% O_2 and 5% CO_2. NACL medium was prepared using the following: 1:1 (v/v) mixture of DMEM/F12, and neurobasal medium, 1 x N2 supplement, 1 x B27 supplement, 1 x GlutaMAX, 1 x nonessential amino acids, 0.1 mM β-mercaptoethanol, 0.5% penicillin/streptomycin, 100 ng/ml activin A, 3 μM CHIR99021 and 10 ng/ml recombinant human LIF.

In-vitro differentiation of iCDX2-ESCs

Differentiation of iCDX2-ESCs into EVTs was performed as previously reported[1, 2]. iCDX2-ESCs culture more than three passages in the presence of doxycycline to generate more mature sheep TSC-like cells，then the cells were seeded at a density of 4 x 10^4 cells per well onto a four-well plate pre-coated with 1 μg/ml collagen IV (Sigma) and cultured in 500 μl EVTs differentiation

medium. On day 3 of differentiation, the medium was replaced with EVT differentiation medium without hNRG1, and Matrigel (Corning) was added to a final concentration of 0.5%. On day 6 of differentiation, EVTs differentiation medium was replaced without hNRG1 and KSR, and Matrigel was added to 0.5% final concentration. For the differentiation of TSCs into STs, iCDX2-ESCs were seeded at a density of 4 x 10^4 cells per well onto a four-well plate pre-coated with 2.5 μg/ml collagen IV (Sigma) and cultured in 500 μl ST differentiation medium. Medium was replaced every other day.

3D suspention in-vitro culture of sheep blastoids and normal sheep blastocysts

The sheep blastoids on day 6 and normal sheep blastocysts on day 8 were manually isolated using a mouth pipette and transferred to new ultra-low attachment plates containing fresh N2B27 basic medium. The plates were placed on a shaker set to 80 rpm during culture. The medium was exchanged every two days. The blastoids and blastocysts were cultured at 37°C, with 20% O_2 and 5% CO_2.

N2B27 basal media was prepared using the following: 1:1 (v/v) mixture of DMEM/F12 and neurobasal medium, 1x N2 supplement, 1 x B27 supplement, 1 x GlutaMAX, 1 x nonessential amino acids, 1x penicillin–streptomycin, 1x Na-pyruvate, 1.6 mg/mL BSA.

Teratoma formation

Sheep ESCs colonies were wash twice by PBS and digested into single cells with TrypLE. One million single cells for each cell line were suspended with a mixture of 100 μLof pre-cooled culture medium and 100 μLof Matrigel and injected subcutaneously into the flank of 6-8 weeks NOD/SCID mice. Teratomas were generally formed in 8-12 weeks. Mice were sacrificed before the teratoma size exceeded 3 cm in diameter. Teratomas were isolated and fixed in 4% paraformaldehyde (PFA), embedded in paraffin, sectioned, and histologically characterized by H&E staining for detecting the presence of representative tissues of all three germ layers.

H&E staining

Teratomas were isolated and fixed in 4% paraformaldehyde (PFA) at 4°C overnight, washed 3 times in DPBS, and then dehydrated by gradient alcohol, which were operated in the dish. The dehydrated teratomas were peeled away from the dish by capillary pipet, vitrified by dimethylbenzene and

embedded in paraffin. Then, the embedded embryos were transversely sectioned, and the sections were stained by H&E staining according to manufacturer's instructions.

Immunofluorescence staining

Cells and embryos were harvested, fixed with 4% (v/v) paraformaldehyde (PFA) at room temperature for 20 minutes, and then washed with 1x PBS. The fixed cells were permeabilized with 1% Triton-X100 at room temperature for 20 minutes, blocked with 3% BSA for 30 minutes, and incubated with primary antibodies overnight at 4°C. After at least three washes, the cells were incubated with fluorescence-conjugated secondary antibodies and 4',6-diamidino-2-phenylindole (DAPI) in the dark at room temperature for 2 hours. Following three washes with 1X PBS, images were captured using a confocal FV3000 microscope. Antibodies used in this study in Supplementary Table S2.

Alkaline phosphatase (AP) staining

AP staining was performed with an Alkaline Phosphatase Detection Kit (Beyotime, C3206) according to the manufacturer's protocol. Briefly, sheep ESCs were seeded on mitomycin c-treated MEF feeders in a well of a 24-well plate and cultured for 4 days in the respective medium. For AP staining, the clones were washed with PBS twice and then incubated with BCIP/NBT working solution for 10-15 minutes in the dark. The solution was washed off and stopped the reaction with ddH_2O. Images of AP^+ clones for different conditions were taken from random fields for analysis and three technical replicates were included.

Real-time quantitative PCR (RT-qPCR)

Total RNA from an entire well of cultured cells was isolated using the TRIZOL reagent (Invitrogen) according to the manufacturer's instructions. RNA was converted to cDNA using T PrimeScript™ RT reagent Kit (RR047A, TaKaRa). Reactions were performed using TB Green® Premix Ex Taq™ II (RR820A, TaKaRa) and run on a LightCycler 480 Instrument II (Roche). Primers are summarized in the Supplementary Table S3.

Karyotype analysis

Karyotyping via G-banding was performed according to a standard protocol. In brief, colchicine (100 ng/mL) was added to the culture of 70-80% confluent sESCs for 1.5 hours to arrest cells at

metaphase. After removing the culture medium, the cells were washed with PBS and then treated with TrypLE for 3 minutes, followed by centrifugation at 1,000 rpm for 5 minutes. Next, pre-warmed hypotonic solution (37°C) was added dropwise to the cell suspension and gently mixed, after which the cells were incubated at 37°C for 30 minutes. A cold fixation solution was then added, and the mixture was kept at room temperature for 10 minutes before centrifuging at 1,000 rpm for 5 minutes. This fixation procedure was repeated three times. Finally, the cell suspension was dropped onto glass slides, air-dried, and stained with Giemsa. Karyotype analysis was performed by examining the G-banded metaphase spreads under a microscope at 100x magnification. For each sample, 20 metaphase spreads were randomly selected and analyzed.

Bulk RNA-seq data analysis

Raw reads were firstly processed through fastp software. In this step, clean reads were obtained by removing reads containing adapter, reads containing poly-N and low-quality reads from raw data. Reference genome (ARS-UI_Ramb_v3.0) and gene model annotation files were downloaded from NCBI database (https://www.ncbi.nlm.nih.gov/datasets/genome/GCF_016772045.2/). Index of the reference genome was built using Hisat2 (v2.0.5) and paired-end clean reads were aligned to the reference genome using Hisat2 (v2.0.5). Gene counts were calculated using featureCounts (v1.5.0-p3) and then FPKM (Fragments Per Kilobase of exon model per Million mapped fragments) was calculated based on the length of gene and read counts mapped to this gene. After removing the genes with zero count in all samples, Differential expression analysis between sESC and sESC-CDX2 was performed using DESeq2 (v1.40.2) R package. Only genes with adjusted $P < 0.05$ & $|\log2\ (\text{fold change})| >= 1$ were defined as significant DEGs and used for further analysis. Heatmaps were generated by ComplexHeatmap (v2.16.0) R package using log2(FPKM+0.1).

Single-cell RNA-seq sequencing

Sheep blastoids with a visible cavity of ICM-like compartment and trophectoderm-like compartment were picked up manually by mouth pipette. Then washed three times with PBS containing 0.1% BSA and dissociated with 0.25% trypsin-EDTA at 37 °C in 5% CO_2 for 10 min. The dissociation was terminated by PBS with 15% FBS and centrifuged the sample at 300g for 5 min. Cell pellets were resuspended in PBS containing 0.05% BSA, filtered through a 40-μm cell strainer and counted. Single-cell suspensions were loaded into a 10X Genomics Chromium Chip

following the manufacturer's instructions 48 (10X Genomics, Chromium Next GEM Single Cell 3′ GEM, Library and Gel Bead Kit v.3.1).

Single-cell RNA-seq data analysis

Raw reads obtained from scRNA-seq experiments were aligned to the sheep genome (ARS-UI_Ramb_v3.0) using the CellRanger pipeline (cellranger-6.1.2, 10X Genomics). The filtered expression matrix with cell barcodes and gene names was loaded with the 'Read10X' function of the Seurat (v.4.3.0) R package. First, cells with detected genes less than 1000 and more than 6000 were removed for further analysis. Next, doublet or multiplet cells were determined with DoubletFinder (v2.0.3) R package according to the recommended multiplet rate reference table from 10X Genomics. Totally, there were 9731 cells (median gene number: 2435) left for downstream analysis. The preprocessed gene expression data of 9731 genes were then analyzed by the Seurat (version 4.3.0) package and the following steps were performed in order: data normalization, highly variable gene selection, principal component analysis (PCA) and clustering. The count matrix was first normalized and scaled. The top 3000 highly variable genes were then obtained by FindVariableGenes function with the default variance stabilizing process. We further embedded ensuing nuclei in the PCA dimensions followed by Uniform Manifold Approximation and Projection (UMAP) visualization. Top 20 principal components were used for cell clustering and the resolution of FindClusters function was set to 0.9. Subsequently, clusters were annotated to lineage based on known lineage markers: *POU5F1* and *SOX2* for ELC, *FOXA2* and *APOB* for HLC, *KRT19* and *NR2F2* for TLC. Genes differentially expressed between lineage (DEGs) were detected by running the "FindAllMarkers" function in Seurat package based on the Wilcox test. Only genes with adjusted P value < 0.05 and log2 fold-change >= 0.3 were selected for further analysis.

Single-cell RNA-seq data collection and integrated analysis

For sc-RNA-seq data of sheep spherical blastocyst (9 dpf), the raw FASTQ reads were aligned to the sheep genome (ARS-UI_Ramb_v3.0) using the CellRanger pipeline (cellranger-6.1.2, 10X Genomics). Cells with detected genes more than 800 were used for further analysis. Firstly, we conducted quality control and data normalization utilizing Seurat (v4.3.0). Secondly, we applied batch effect removal and data embedding to all cells using the harmony algorithm, generating a UMAP that visualizes the integration of sheep blastoid and sheep spherical blastocyst.

For scRNA-seq data of sheep blastocyte (E7-8), data preprocessing was performed as described in the original study[3]. Briefly, umi_tools (v1.1.6) [4]was used to demultiplex the data via the 8-bp cell barcodes and 8-bp unique molecular identifiers (UMIs) in Read 2, with cell-specific barcode sequences retrieved from the original study's supplementary table. Kb-python (v0.30.0) [5]was then used for read alignment and UMI counting against the sheep genome (ARS-UI_Ramb_v3.0). The count matrix and related metadata were imported into Seurat (v4.3.0) for data normalization, highly variable gene selection, PCA and clustering. Cell types were annotated based on lineage specific markers.

For the integrated analysis among three datasets (sheep blastoid D6, spherical blastocyst(D9_SB) and E7-8 blastocyte), Seurat objects were constructed using 16,457 shared genes. Data integration was then performed via the LIGER_integrate function in the SCP package (v0.5.6) with parameters: batch=" study", nonlinear_reduction="umap". The AverageExpression function (Seurat v4.3.0) was used to calculate average gene expression per cell type.

Gene sets scoring

We used ''AddModuleScore'' function in Seurat package to calculate the gene sets scores. The sheep blastocyst lineage-specific scores were calculated using the DEGs of each lineage in the sheep spherical blastocyst [6].

Functional enrichment analysis

GO enrichment analyses were conducted using g: Profiler and the significant GO terms with FDR-corrected $p < 0.05$ were reported.

Statistical analysis

Comparison between two groups was analyzed by unpaired two-tailed Student's t-test and comparison among three to four groups was analyzed by one-way ANOVA test. GraphPad Prism 6 software was used for analyzing data. Packages ggplot2 (v3.4.4) and ComplexHeatmap (v2.16.0) were used to prepare the figures. Software and Algorithm used in this study in Supplementary Table S4.

Supplementary Figures and Figure lengends

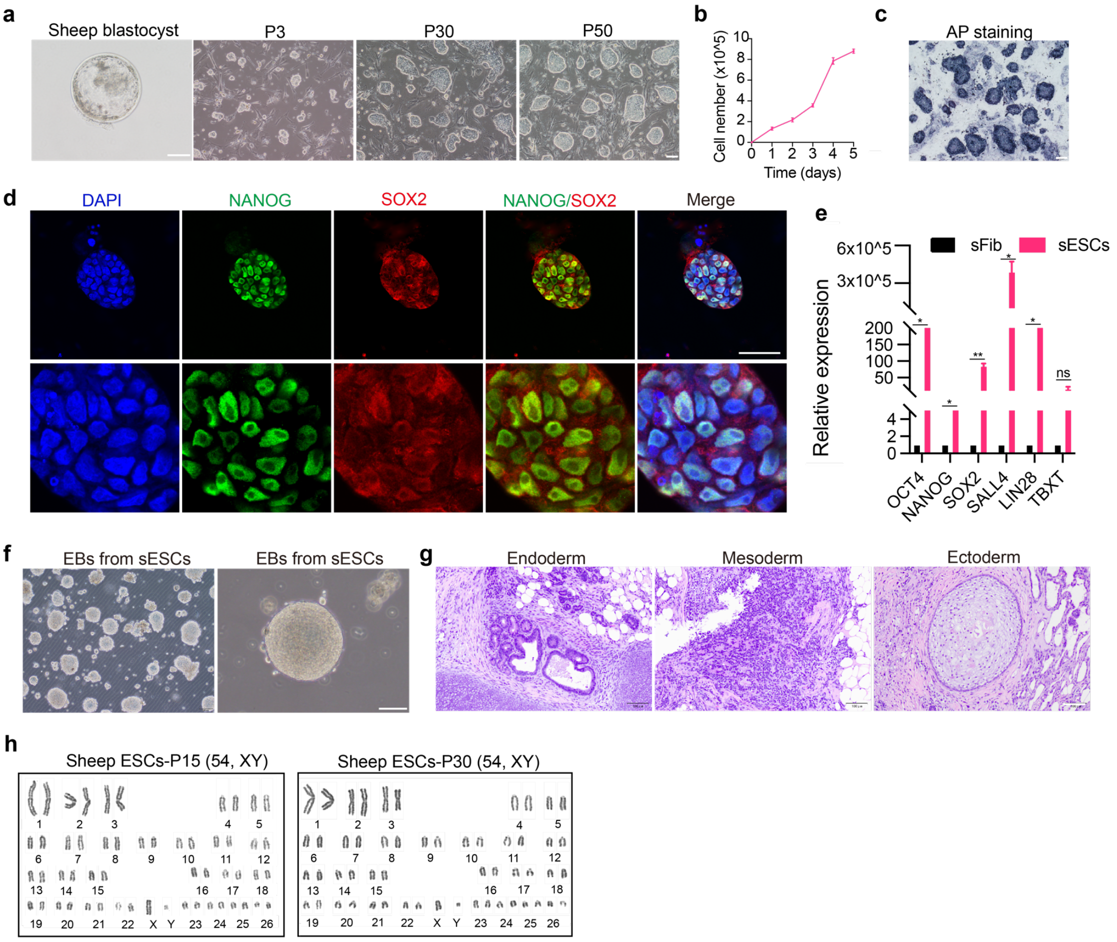

Fig S1. Characterization of sheep ESCs cultured.

(a) Representative phase contrast images showing the derivation of sheep ESCs from blastocysts. Scale bars, 100 μm. P, passage.

(b) Cell proliferation data. The densities of seeded cells (2 x 10^4 cells/well).

(c) Representative images showing AP^+ ESCs colonies after single-cell disassociation. Scale bar, 100 μm.

(d) Representative immunofluorescence images for NANOG (green) and SOX2 (red) in sheep ESCs. Nuclei were counterstained with DAPI. Scale bar, 50 μm.

(e) RT-qPCR for the pluripotency genes in sESCs. Data are presented as mean ± SEM of fold-change compared to primed ESCs. * $p < 0.05$, ** $p < 0.01$, *** $p < 0.001$. n=3 biological replicates.

(f) Represent the morphological of embryoid body formation from sESCs. Scale bar, 100 μm.

(g) H&E staining of teratomas. Representative images of the three germ layers are shown. Scale bar, 100 μm.

(h) Representative images of G-banding karyotype of sESCs.

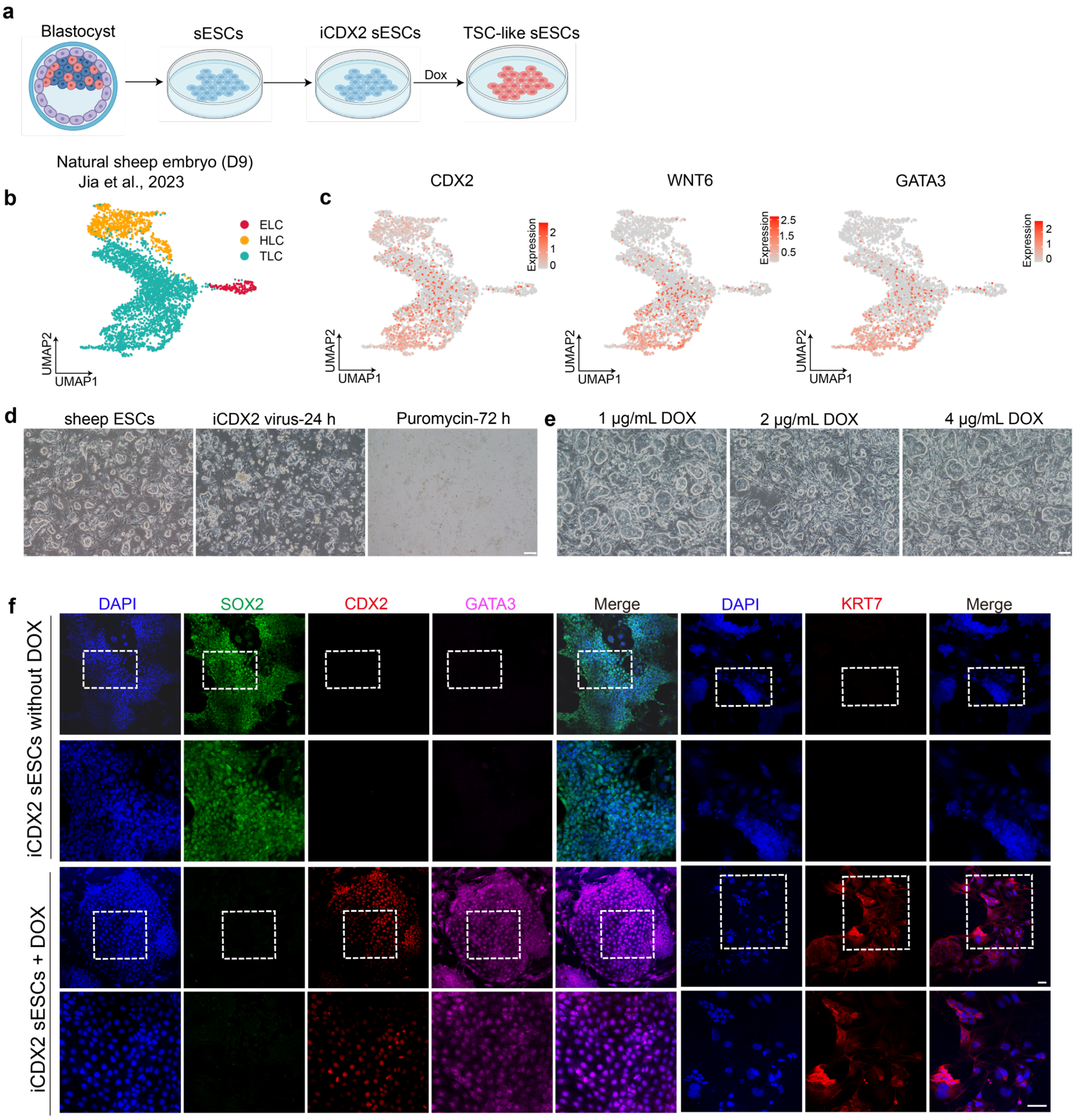

Fig S2. Characterization of trophoblast-like cell induction.

(a) Schematic illustrating the procedure for generating inducible overexpression of *CDX2*.

(b) UMAP plot depict the clustering of cells in D9 natural sheep embryos. ELC, EPI-like cells; HLC, hypoblast-like cells; TLC, trophectoderm-like cells.

(c) UMAP showing expression of TLC markers (*CDX2*, *WNT6* and *GATA3*). The color gradient from gray to red indicates an increase in gene expression, ranging from low to high.

(d) Representative images showing the generation of overexpression *CDX2* sESCs lines. Scale bars, 100 μm.

(e) Representative phase contrast images showing overexpression *CDX2* sESCs colony morphology. Doxycycline was added at concentrations of 1 μg/mL, 2 μg/mL, 4 μg/mL for 4 days. Scale bar, 100 μm. P, passage.

(f) Representative images of immunofluorescent staining of sheep iCDX2 ESC (12 days of doxycycline addition in E8+K medium, doxycycline was added at concentrations of 1 μg/mL). Scale bars, 100 μm.

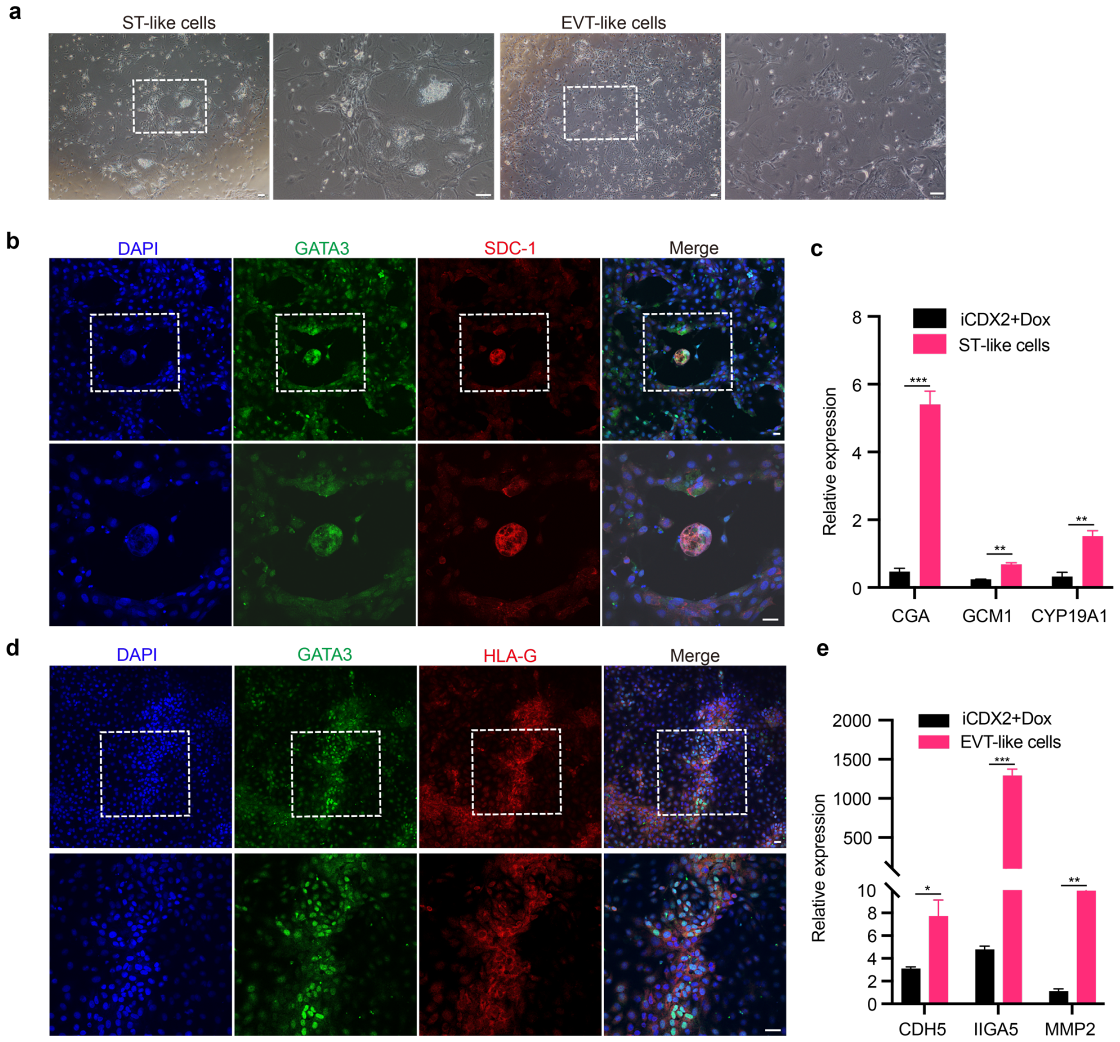

Fig S3. Derivation of EVT-like cells and ST-like cells from sheep iCDX2-ESCs

(a) Representative images showing differentiation of EVT-like cells and ST-like cells from sheep iCDX2-ESCs. Scale bars, 100 µm. EVT, extravillous cytotrophoblasts; ST, syncytiotrophoblasts.

(b) Representative immunofluorescent staining images of marker genes detection in ST-like cells (SDC1/GATA3). Scale bars, 25 µm.

(c) Real-time qPCR results showing the relative expression levels of marker genes of ST-like cells (*CGA, GCM1, CYP19A1*). Data are presented as mean ± SEM of fold-change. * $p < 0.05$, ** $p < 0.01$, *** $p < 0.001$. The P values were calculated using unpaired t-tests. n=3 biological replicates.

(d) Representative immunofluorescent staining images of marker genes detection in EVT-like cells (HLA-G/GATA3). Scale bars, 25 µm.

(e) Real-time qPCR results showing the relative expression levels of marker genes of EVT-like cells (*CDH5, IIGA5, MMP2*). Data are presented as mean ± SEM of fold-change. * $p < 0.05$, ** $p < 0.01$, *** $p < 0.001$. The P values were calculated using unpaired t-tests. n=3 biological replicates.

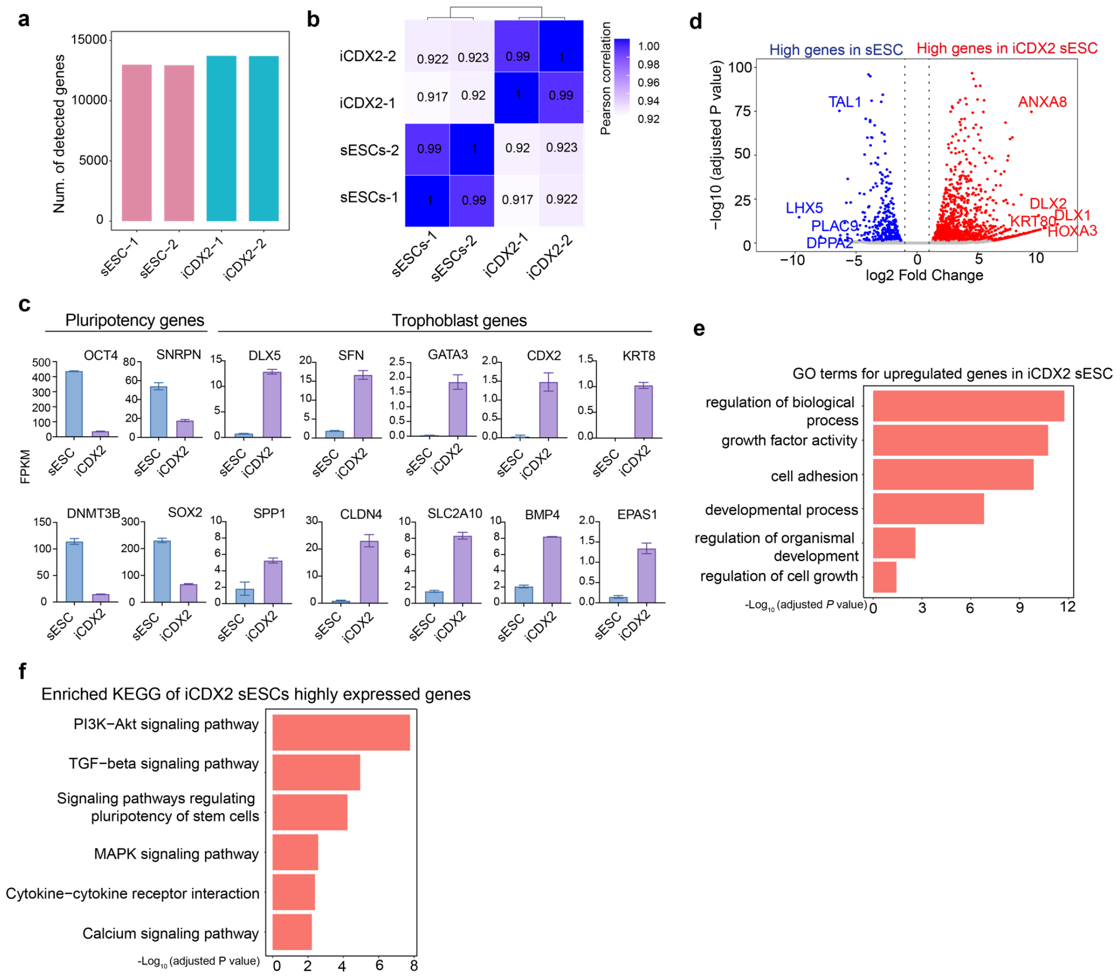

Fig S4. Transcriptome characterization of *CDX2* overexpression sESCs.

(a) Bar plot showing the number of detected genes in sheep ESCs and iCDX2 sESCs.

(b) Heatmap showing the correlation between samples of sESCs and iCDX2 sESCs.

(c) Bar plots showing the expression patterns of classic pluripotency genes and trophectoderm genes in sESCs and iCDX2 sESCs.

(d) Volcano plot showing DEGs between sheep sESCs and iCDX2 sESCs. DEGs higher in iCDX2 sESCs (log2(fold change) > 1) are shown in red.

(e) Enriched GO terms for upregulated genes in iCDX2 sESCs. P value was calculated using a Fisher's one-tailed test and adjusted for multiple testing using the Benjamini-Hochberg correction.

(f) Enriched KEGG pathways for upregulated genes in iCDX2 sESCs.

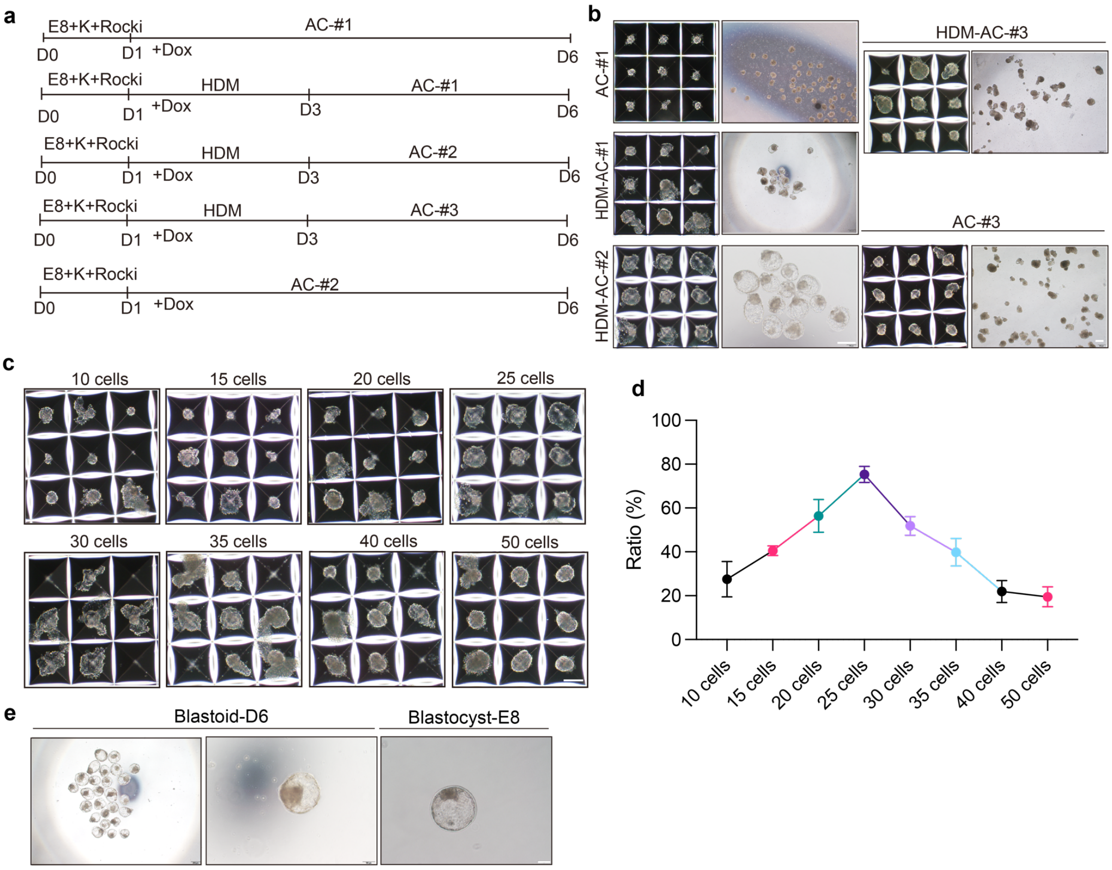

Fig S5. Optimized procedure for producing sheep blastoid.

(a) Schematic showing induces blastoid using different protocols.

(b) Representative images showing the morphology of blastoid using different protocols. Scale bars: 200 μm.

(c) Representative images showing the morphology of induces blastoid different starting cell numbers. Scale bars: 200 μm.

(d) Ratio of blastoids to all aggregates generated from different starting cell numbers.

(e) Representative images of sheep blastoids and natural sheep blastocyst. Scale bars, 50 μm.

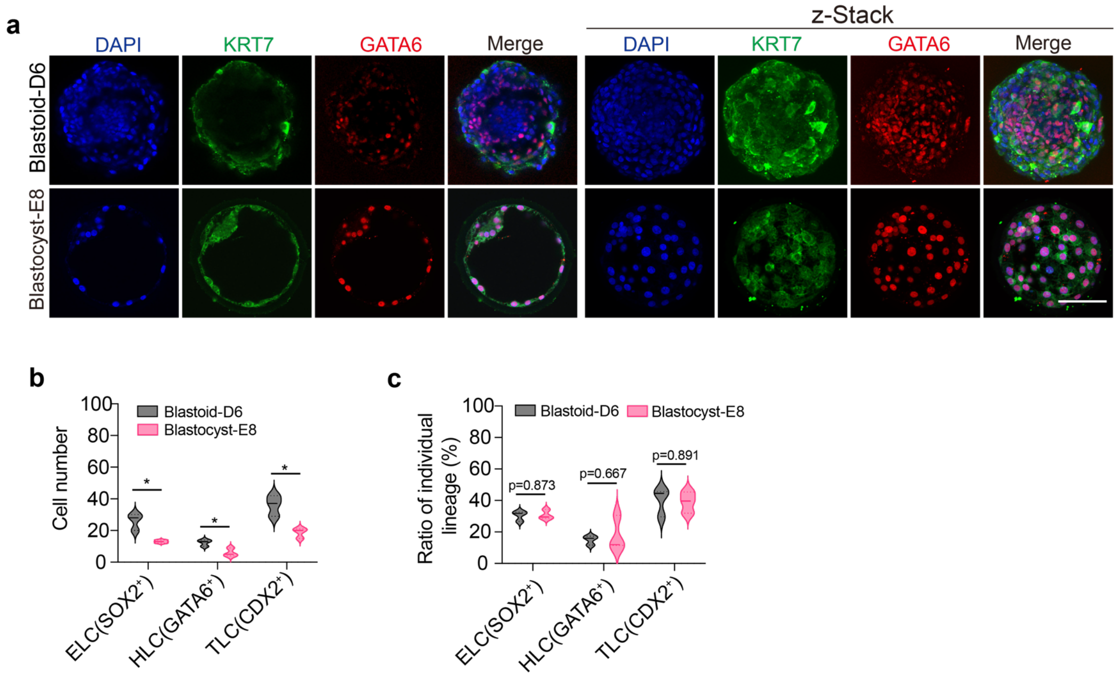

Fig S6. Immunofluorescent staining of sheep blastoids.

(a) Representative immunofluorescent staining of trophectoderm (KRT7) and hypoblast (GATA6) in reconstructed sheep blastoids on D6 and blastocyst. Scale bars, 50 µm.

(b) Total cell numbers of per blastoid (n = 3 biological replicates) and sheep blastocyst (n = 3 biological replicates). Data are presented as mean ± SEM of fold-change. * $p < 0.05$, ** $p < 0.01$, *** $p < 0.001$. The P values were calculated using unpaired t-tests.

(c) Ratio of individual lineage of per blastoid (n = 3 biological replicates) and sheep natural blastocyst (n = 3 biological replicates). Data are presented as mean ± SEM of fold-change. * $p < 0.05$, ** $p < 0.01$, *** $p < 0.001$. The P values were calculated using unpaired t-tests.

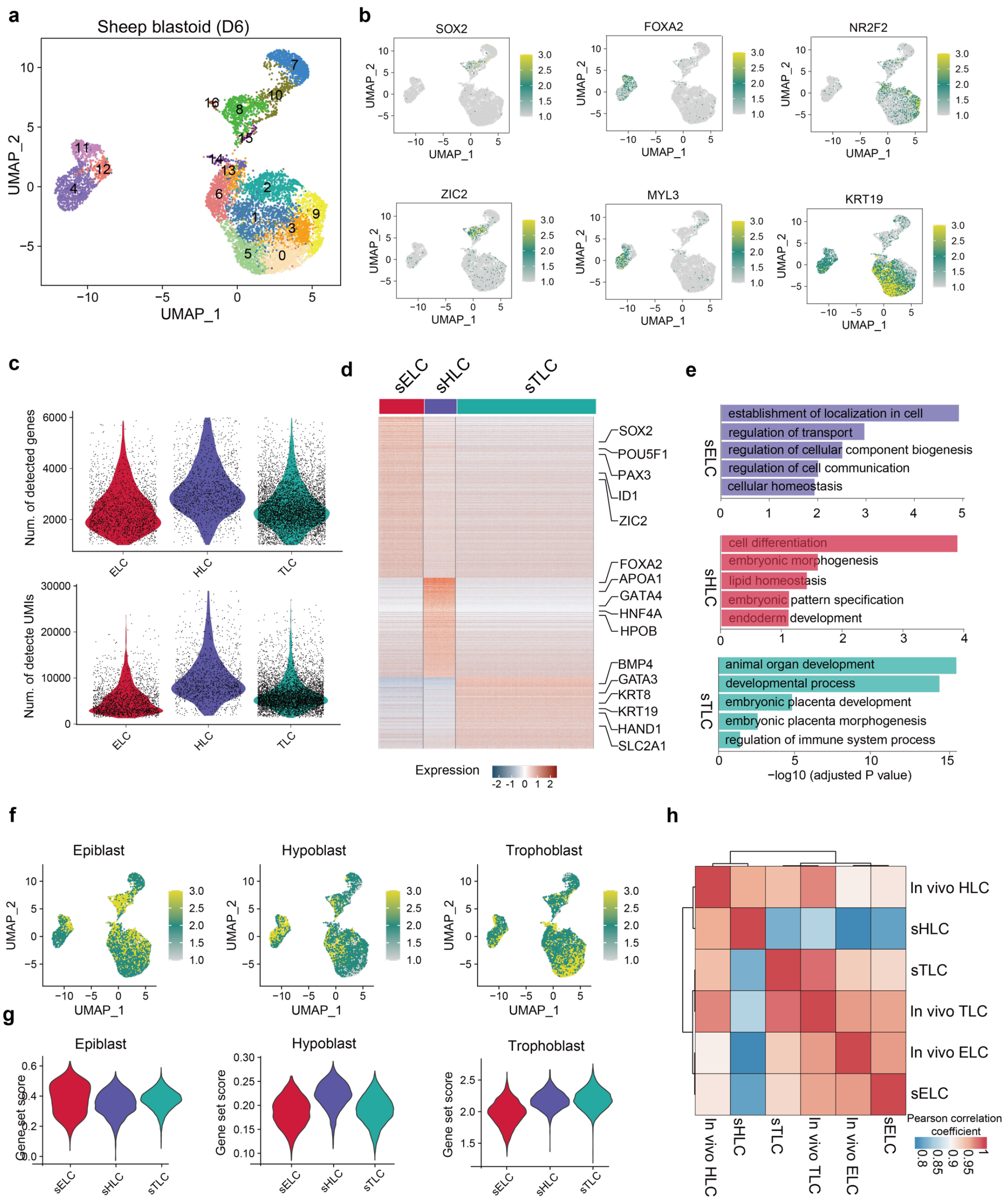

Fig S7. Single-cell RNA-seq characterization of sheep blastoids.

(a) UMAP plot showing the clustering of cells from sheep blastoid, colored according to distinct clusters.

(b) UMAP showing expression of ELC markers (*SOX2, ZIC2*), HLC markers (*FOXA2, MYL3*), and TLC markers (*NR2F2, KRT19*) in sheep blastoid.

(c) Violin plots showing the number of detected genes and UMIs in major cell lineages.

(d) Heatmap representing the expression of cell lineage-specific genes in ELC, HLC, and TLC.

(e) UMAP plots displaying the sheep blastocyst lineage-specific signature scores in sheep blastoid cells.

(f) Violin plots displaying the sheep blastocyst lineage-specific signature scores in sheep blastoid lineages.

(g) Bar plots showing the represented GO terms of cell lineage-specific genes in sELC, sHLC, and sTLC. The x axis represents the adjusted P value.

(h) Heatmap showing the similarity among major cell lineages in sheep blastoid and nature sheep blastocyst (D9_SB).

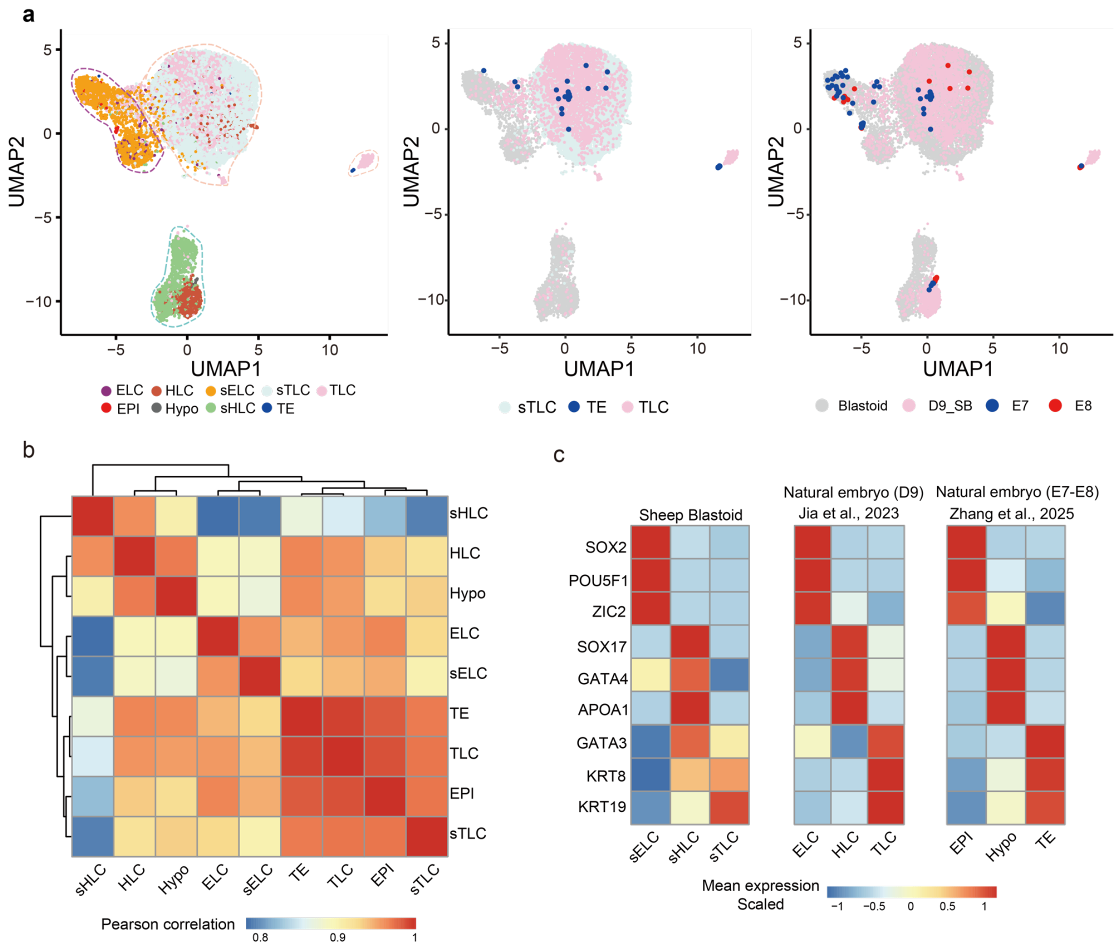

Fig S8. Integrated analysis of cells from sheep blastoids at D6, spherical blastocytes at 9 days post fertilization (9 dpf), and natural sheep blastocytes at E7-E8.

(a) UMAP plots depict the clustering of cells from sheep blastoids at D6, spherical blastocytes at 9 dpf, and natural sheep blastocytes at E7-E8. ELC, EPI-like cells; HLC, hypoblast-like cells; TLC, trophectoderm-like cells.

(b) Heatmap shows clusters of the same lineage exhibit high transcriptomic similarity.

(c) Heatmap shows the similar expression pattern of lineage specific genes in sheep blastoids at D6, spherical blastocytes at 9 dpf, and natural sheep blastocytes at E7-E8.

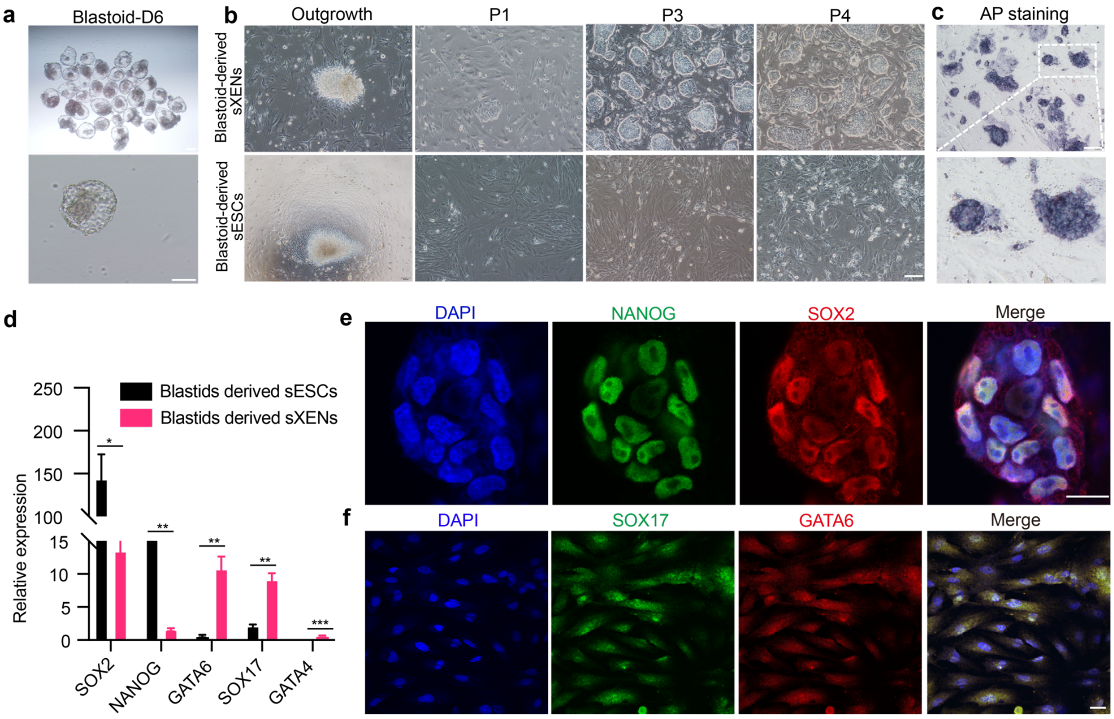

Fig S9. Stem cells derivation from sheep blastoids.

(a) Representative images of sheep blastoids at D6. Scale bars, 50 µm.

(b) Representative phase contrast images showing the derivation of sheep ESCs and sheep extraembryonic endoderm (sXENs) from blastoids. Scale bars, 100 µm. P, passage.

(c) AP staining of sESCs derived from blastoids. Scale bars, 100 µm.

(d) Real-time qPCR results showing the relative expression levels of marker genes of ESC (*SOX2, NANOG*) and sXEN (*GATA6/SOX17/GATA4*). (n=3 for each group). Data are presented as mean ± SEM of fold-change. * $p < 0.05$, ** $p < 0.01$, *** $p < 0.001$. The P values were calculated using unpaired t-tests.

(e) Representative immunostaining of SOX2 and NANOG at the derivation of sESCs from blastoids. Nuclei were counterstained with DAPI (blue). Scale bar, 50 µm.

(f) Representative immunofluorescent staining images of sXENs. Scale bars, 25 µm.

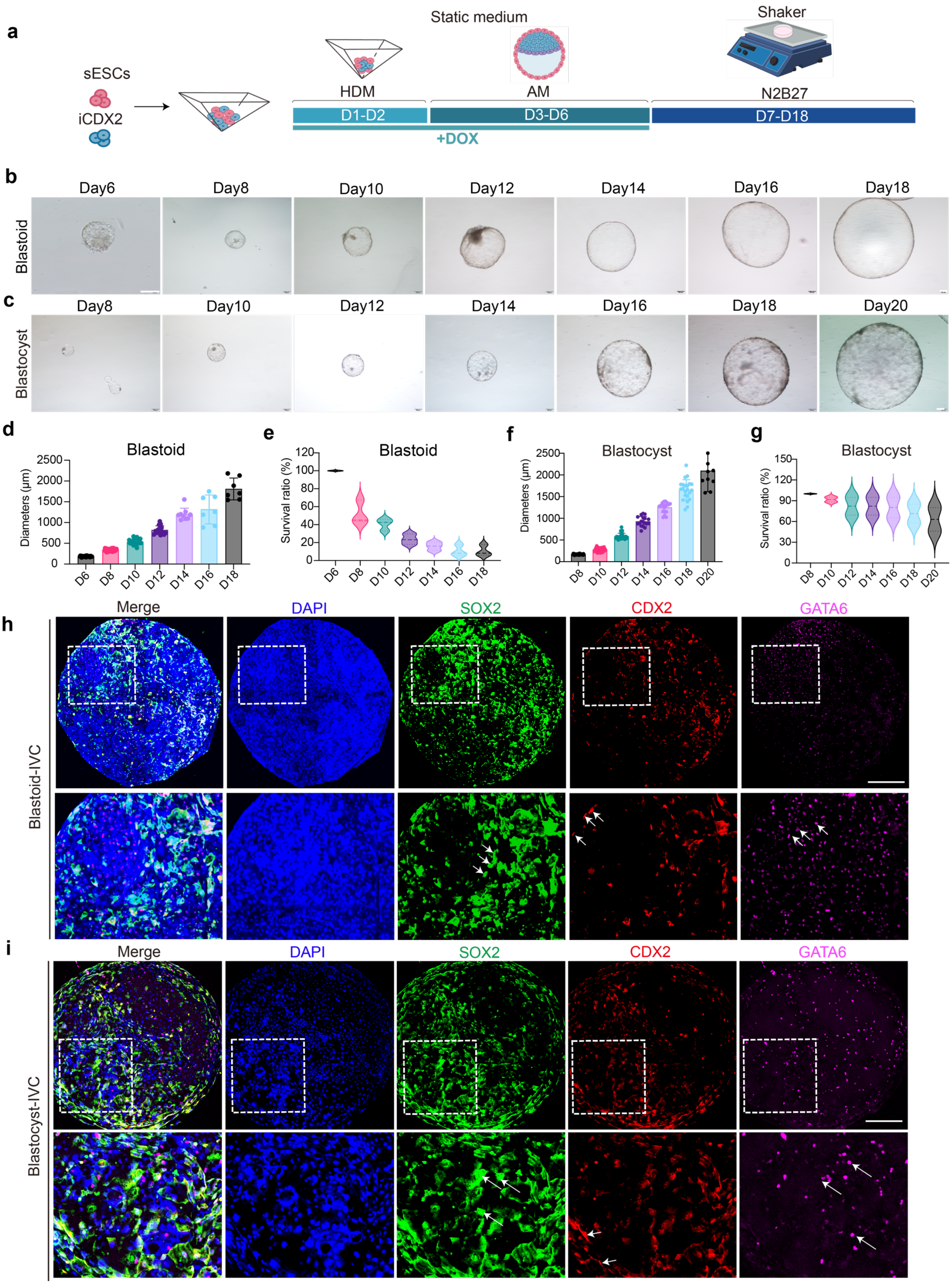

Fig S10. In-vitro-cultured of sheep blastoids.

(a) Schematic depiction of the blastoids generation and culture protocol. Doxycycline induction and aggregation of two types of followed by culture in HDM and AM (with Dox for 6 days), N2B27 medium for 11 days.

(b) The dynamic of the morphology of in vitro cultured blastoids from day 6 to day 18. Scale bars, 200 μm.

(c) The dynamic of the morphology of normal sheep embryo from day 8 to day 20. Scale bars, 200 μm.

(d-j) The diameters and survival ratio of *in vitro* cultured blastoids and normal sheep embryo without fragmentation or pyknosis.

(h-i) Representative immunofluorescence images of sheep blastoids and normal sheep embryos cultured for an additional 12 days in prolonged culture medium. Scale bars, 100 μm.

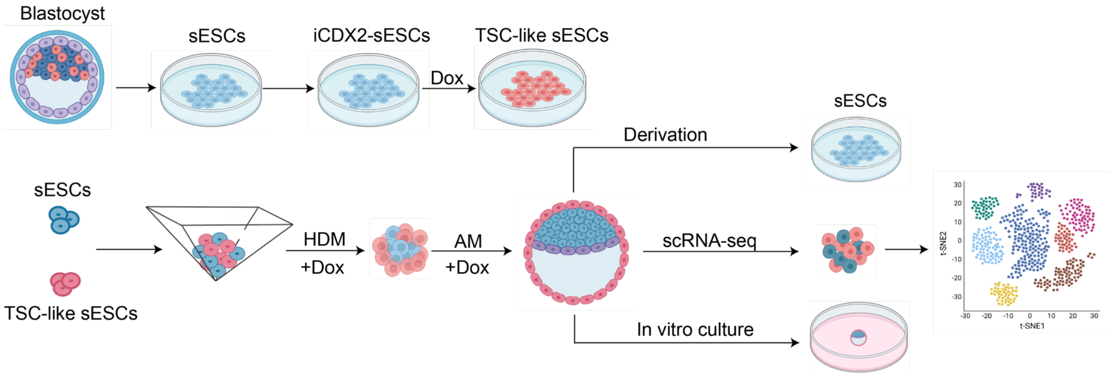

Fig S11. A diagram summary of the major findings of this study.

Supplementary Table S1 Reagents used in this study.

Reagents	Source	Catalog
TrypLE Express	Thermo Fisher Scientific	Cat# 12604021
0.25% Trypsin-EDTA	Meilunbio	Cat# MB4376
Accutase	Sigma	Cat# A6964
Collagenase IV	Thermo Fisher Scientific	Cat# 17104019
Neurobasal medium	Thermo Fisher Scientific	Cat# 21103049
DMEM/F12	Thermo Fisher Scientific	Cat# 10565018
Glutamax	Thermo Fisher Scientific	Cat# 35050061
TeSR-E8 Kit for hESC/hiPSC	Stem cell	Cat# 05990
mTeSR-Plus basal medium	Stem cell	Cat# 100-0274
FBS	BI	Cat# 04-001-1ACS
KOSR	Thermo Fisher Scientific	Cat# A3181502
Nonessential amino acids	Thermo Fisher Scientific	Cat# 11140050
Penicillin and streptomycin	Thermo Fisher Scientific	Cat# 15140122
Sodium Pyruvate	Thermo Fisher Scientific	Cat# 129830-382
N2	Thermo Fisher Scientific	Cat# 17504044
B27	Thermo Fisher Scientific	Cat# 17502048
Matrigel	Corning	Cat# 354277
BSA	ABCONE	Cat# A23088
IWR-1	Sigma	Cat# I0161
MEK inhibitor PD0325901	Axon	Cat# 1408
Activin/nodal receptor inhibitor A83-01	Sigma	Cat# SML0788
ITS-X	Thermo Fisher Scientific	Cat# 51500056
L-ascorbic acid	Sigma	Cat# A4030
EGF	PeproTech	Cat# AF-100-15-100
CHIR99021	Sigma	Cat# SML1046
SB431542	Selleck	Cat# S1067
Valproic acid	Stem cell	Cat# 72292
GELTREX LDEV free hESC quality	Thermo Fisher Scientific	Cat# A1413302

Y27632	Axon	Cat# 1683
Mitomycin C	Sigma	Cat# M0503-5
Opti-MEM medium	Thermo Fisher Scientific	Cat# 31985070
Doxycycline	Sigma	Cat#324385
BCIP/NBT Alkaline Phosphatase Color Development Kit	Beyotime	Cat# C3206
Lipofectamine 3000 Transfection Reagent	Invitrogen	Cat# L3000001
PrimeScript™ RT reagent Kit	TaKaRa	Cat# RR047A
TB Green® Premix Ex Taq™ II	TAKARA	Cat# RR820A

Supplementary Table S2 Antibodies used in this study.

Name	Source	Catalog	Dilution
Rabbit anti-CDX2	Abcam	Cat# ab76541;	1:200
Rabbit anti-GATA6	CST	Cat# 5851;	1:200
Mouse anti-SOX2	Santa Cruz	Cat# sc365823	1:200
Rabbit anti-NANOG	CST	Cat# 4903;	1:500
Mouse anti-KRT7	Dako	Cat# 20064396	1:100
Mouse anti-ZO-1	Invitrogen	Cat# 339100;	1:500
Goat anti-SOX17	R&D systems	Cat# AF1924	1:500
Rabbit anti-GATA6	CST	Cat# 5851	1:500
Rabbit anti-HLA-G	Abcam	Cat# ab283260	1:500
Rabbit anti-SDC1	Abcam	Cat# ab128936	1:500
Goat anti-GATA3	R&D systems	Cat# AF2605	1:500
Alexa Fluor 488-AffiniPure Donkey Anti-Mouse IgG (H+L)	Jackson ImmunoResearch Labs	Cat# 715-545-151;	1:1000
Alexa Fluor 488-AffiniPure Donkey Anti-Rabbit IgG (H+L)	Jackson ImmunoResearch Labs	Cat# 711-545-152;	1:1000
Alexa Fluor 647-AffifiniPure Donkey Anti-Goat IgG (H+L)	Jackson ImmunoResearch Labs	Cat# 705-605-147;	1:1000
Alexa Fluor CY3-AffiniPure Donkey Anti-Mouse IgG (H+L)	Jackson ImmunoResearch Labs	Cat# 715-165-150;	1:1000
Alexa Fluor CY3-AffiniPure Donkey Anti-Rabbit IgG (H+L)	Jackson ImmunoResearch Lab	Cat# 705-165-147;	1:1000
DAPI	Sigma	Cat# D9542	1:1000

Supplementary Table S3 Primers for real-time quantitative PCR analyses used in this study.

Gene name	Sequence
Sheep GAPDH primer-Forward	GTTCCACGGCACAGTCAAGG
Sheep GAPDH primer-Reverse	ACTCAGCACCAGCATCACCC
Sheep OCT4 primer-Forward	AACGAGAATCTGCAGGAGATATG
Sheep OCT4 primer-Reverse	TCTCACTCGGTTCTCGATACT
Sheep NANOG primer-Forward	CAGCTACAAGCAGGTGAAGA
Sheep NANOG primer-Forward	CTATTCCTCGGCCAGTTGTT
Sheep OCT4 primer-Reverse	CATTAACGGCACACTGCCCC
Sheep SOX2 primer-Reverse	TGAAAATGTCTCCCCCGCCC
Sheep SALL4 primer-Forward	TTAGACCCGTCCAAGAAAGGC
Sheep SALL4 primer-Reverse	GTGGATCTGCAAGGAGCTATCA
Sheep TBXT primer-Forward	TGAAGGTGAACGTATCCGGC
Sheep TBXT primer-Reverse	CTCCCCGTTCACGTACTTCC
Sheep LIN28 primer-Forward	ATGGGCTCTGTGTCAAACCA
Sheep LIN28 primer-Reverse	TGCGCACGTTGAACCACTTA
Sheep CDX2 primer-Forward	TGGGCAGCCAAGTGAAA
Sheep CDX2 primer-Reverse	CTTTCCTCCGGATGGTGATATAG
Sheep GATA3 primer-Forward	CCACCTACCCACCATACGTC
Sheep GATA3 primer-Reverse	CGGTTCTGTCCGTTCATCTT
Sheep ASCL2 primer-Forward	ACCCAAGGCTAGTGTGCAAG
Sheep ASCL2 primer-Reverse	CGTCGTCATAAAGCCCTCTC
Sheep KRT8 primer-Forward	TACGGGACCCCTGGCTTCAACTAC
Sheep KRT8 primer-Reverse	CATCGCGGGTCTCAATCTTCTTCA
Sheep CGA primer-Forward	GGGTTGTCCTGAATGCAAGC
Sheep CGA primer-Reverse	CAGCACCCCATGCACTGATA
Sheep GCM1 primer- Forward	CTCCGGGGCTTTAACGTTTCC
Sheep GCM1 primer-Reverse	AATCCGCGTTCTCAGGTTCC
Sheep CYP19A1 primer- Forward	CCGAAGTTGTGCCTATTGCC
Sheep CYP19A1 primer-Reverse	GCTGGGACCTGGTATTGAGG
Sheep CDH5 primer- Forward	GGAACAGATACACTGTGAGCC
Sheep CDH5 primer-Reverse	GTACTCGGTGTTCTTGCGGT
Sheep ITGA5 primer- Forward	CTCAGTCTCTCCTCGTCCCA
Sheep ITGA5 primer-Reverse	TGACTGGTGGTGCAGTTGTT
Sheep MMP2 primer- Forward	GGACAAGTGGTCCGTGTCAA
Sheep MMP2 primer-Reverse	GCTGTCGTAAGATGTGCCCT
Sheep GATA6 primer- Forward	ACCCGAGTCTCAGGAGCTAA
Sheep GATA6 primer-Reverse	AGCAGGAGGAAGAGGAGGAG
Sheep SOX17 primer- Forward	GGCATCTCCTGTCTCGCT
Sheep SOX17 primer-Reverse	CGTTGTGCAGATCTGGGTTCT
Sheep GATA4 primer- Forward	CCCCCTTGGTGATCCCTTC
Sheep GATA4 primer-Reverse	GTTCAAGCAGGAAGCCGAATC

Supplementary Table S4 Software and Algorithm used in this study.

Software name	Source	website
Fiji-ImageJ	National Institute of Health	https://imagej.net/Fiji; RRID:SCR_003070
GraphPad Prism 6	GraphPad	https://www.graphpad.com/scientific-software/prism/; RRID:SCR_002798
fastp (v0.20.1)	Chen et al., [4]	https://github.com/OpenGene/fastp
Hisat2 (v2.0.5)	Kim, D. et al., [5]	https://github.com/DaehwanKimLab/hisat2
featureCounts (v1.5.0-p3)	Liao et al.,[6]	https://subread.sourceforge.net/featureCounts.html
Cell Ranger (v6.1.2)	10X Genomics	https://www.10xgenomics.com/support/software/cell-ranger/downloads
umi_tools	Simth, S.T. et al. 2017	https://github.com/CGATOxford/UMI-tools
kb-python	Sullivan, D.K. et al., 2025	https://github.com/pachterlab/kb_python
R (v4.3.2)	R Core Team	https://www.R-project.org
Seurat (v4.3.0)	Satija Lab	https://satijalab.org/seurat/
DoubletFinder (v2.0.3)	McGinnis et al., [7]	https://github.com/chris-mcginnis-ucsf/DoubletFinder
ggplot2 (v3.4.4)	Wickham, 2016	https://cran.r-project.org/web/packages/ggplot2/index.html
SCP	**Hao Zhang**	https://github.com/zhanghao-njmu/SCP
DESeq2 (v1.40.2)	Love MI et al.,[8]	https://bioconductor.org/packages/release/bioc/html/DESeq2.html
ComplexHeatmap (v2.16.0)	Gu, et al., [9]	https://github.com/jokergoo/ComplexHeatmap
gprofiler2 (v0.2.3)	Kolberg L, [10]	https://cran.r-project.org/web/packages/gprofiler2/index.html

Supplementary Table S5: The list and enriched functional terms of differential expression genes between sESC and iCEDX2 sESCs. Related to Figure 1.

Supplementary Table S6: Identified cell types and corresponding marker genes and enriched functional terms of lineage-specific genes of sheep D6 blastoid. Related to Supplementary Figures S7.

Reference

1. Yu, L. *et al.* Blastocyst-like structures generated from human pluripotent stem cells. *Nature* **591**, 620-626 (2021).
2. Okae, H. *et al.* Derivation of Human Trophoblast Stem Cells. *Cell stem cell* **22**, 50-63 e56 (2018).
3. Zhang, J. *et al.* Tracing and Capturing the Epiblast Pluripotency of Sheep Preimplantation Embryos. *Adv Sci (Weinh)*, e17764 (2025).
4. Smith, T., Heger, A. & Sudbery, I. UMI-tools: modeling sequencing errors in Unique Molecular Identifiers to improve quantification accuracy. *Genome research* **27**, 491-499 (2017).
5. Sullivan, D.K. *et al.* kallisto, bustools and kb-python for quantifying bulk, single-cell and single-nucleus RNA-seq. *Nature protocols* **20**, 587-607 (2025).
6. Jia, G.X. *et al.* Single-cell transcriptomic characterization of sheep conceptus elongation and implantation. *Cell reports* **42**, 112860 (2023).
7. Chen, S., Zhou, Y., Chen, Y. & Gu, J. fastp: an ultra-fast all-in-one FASTQ preprocessor. *Bioinformatics* **34**, i884-i890 (2018).
8. Kim, D., Paggi, J.M., Park, C., Bennett, C. & Salzberg, S.L. Graph-based genome alignment and genotyping with HISAT2 and HISAT-genotype. *Nature biotechnology* **37**, 907-915 (2019).
9. Liao, Y., Smyth, G.K. & Shi, W. featureCounts: an efficient general purpose program for assigning sequence reads to genomic features. *Bioinformatics* **30**, 923-930 (2014).
10. McGinnis, C.S., Murrow, L.M. & Gartner, Z.J. DoubletFinder: Doublet Detection in Single-Cell RNA Sequencing Data Using Artificial Nearest Neighbors. *Cell Syst* **8**, 329-337 e324 (2019).
11. Love, M.I., Huber, W. & Anders, S. Moderated estimation of fold change and dispersion for RNA-seq data with DESeq2. *Genome biology* **15**, 550 (2014).
12. Gu, Z., Eils, R. & Schlesner, M. Complex heatmaps reveal patterns and correlations in multidimensional genomic data. *Bioinformatics* **32**, 2847-2849 (2016).
13. Kolberg, L., Raudvere, U., Kuzmin, I., Vilo, J. & Peterson, H. gprofiler2 -- an R package for gene list functional enrichment analysis and namespace conversion toolset g:Profiler. *F1000Res* **9** (2020).
